# Beyond Prescribing: GLP-1 Care and Obesity Prevention in Primary Care

**DOI:** 10.1177/21501319261494129

**Published:** 2026-09-25

**Authors:** Waseem Jerjes, Elisa Pineda, Austen El-Osta, Azeem Majeed

**Affiliations:** 1 School of Public Health, Faculty of Medicine, 4615 Imperial College London, London, UK

**Keywords:** obesity, GLP-1 receptor agonists, primary care, prevention policy, obesogenic environments, health equity

GLP-1-based therapies have changed obesity care, but they have not changed the social, commercial and built conditions that make obesity common. Semaglutide and tirzepatide can deliver substantial weight loss and cardiometabolic benefit for selected patients, and primary care should not dismiss them as lifestyle shortcuts. Yet their visibility creates a policy risk: medicines may be treated as the most visible sign of obesity action while food, activity and local-environment policy remain weak. We propose the Integrated Obesity Response Framework, a practical way to judge whether GLP-1 expansion is embedded in a wider obesity strategy rather than left as a stand-alone treatment channel. The framework does not ask patients to wait for perfect prevention. It argues that treatment, targeted prevention and population prevention should be strengthened together, with primary care supported across the whole continuum.^1-4^

## A therapeutic breakthrough with a public health risk

Obesity is a chronic, relapsing disease shaped by biology, social conditions, commercial determinants and physical environments; it is not reducible to appetite or willpower. The clinical case for better treatment is therefore strong. Weekly semaglutide and tirzepatide have raised the therapeutic ceiling, with trial results showing weight loss that was previously associated mainly with bariatric surgery, alongside evidence of cardiovascular benefit in high-risk adults without diabetes.^5-20^ For patients living with severe obesity, metabolic risk, pain, immobility, mental ill health, polypharmacy or repeated unsuccessful attempts at weight reduction, these medicines can be life changing. A serious primary care response should therefore protect access for those most likely to benefit.

No patient already living with obesity should be asked to wait for upstream policy to improve. Effective pharmacotherapy may reduce current risk, improve function and lower future demand for some downstream services, even though the size of these effects will vary by comorbidity and by the quality of follow-up. The distinction made in this paper is therefore not obesity care versus prevention. It is between a coordinated obesity strategy and a narrower treatment-only response.

The ‘Obesity First’ approach, advocated in US clinical perspectives, prioritises treating obesity itself rather than managing only its downstream complications. It encompasses nutritional and behavioural support, physical activity, medicines and metabolic surgery where appropriate, rather than pharmacotherapy alone.^21^ This clinical priority is compatible with the Integrated Obesity Response Framework. However, preventing complications in people already living with obesity is not the same as preventing obesity across a population. Earlier treatment should therefore complement, not displace, measures addressing unhealthy food environments, inactivity and unequal exposure. An integrated response supports both timely care for people living with obesity and sustained action to reduce the conditions that produce it.

However, clinical success is not the same as public health success. By randomised withdrawal studies, we mean trials in which participants who have received active treatment are randomly assigned either to continue it or to stop it, so that the durability of benefit can be assessed. Maintenance studies similarly test whether continuing treatment sustains or adds benefit over time. These studies show that weight regain is common when therapy stops, while continued treatment maintains or augments benefit.^6,7,12,22-33^ This is not a weakness of the medicines; it is a reminder that obesity is a chronic relapsing condition. The concern is what happens when a chronic disease requiring long-term treatment is produced within environments that continue to promote excess intake. Pricing and supply will also change. Recent Canadian authorisations of generic semaglutide and the Indian regulator’s warning after multiple generic GLP-1 variants entered the market show that health systems cannot rely on high prices, limited supply or specialist-only routes to contain demand.^34,35^ If access expands faster than pathways, workforce, prevention policy and safety oversight, prescribing may grow faster than the system’s ability to support it well. If access expands while prevention remains weak, prescribing may grow faster than the political will to change food systems.

The risk is amplified by the way obesity debates are communicated. Medicines are tangible, nameable and easy to count. Food-environment reform is diffuse, contested and slower to claim credit for.^26^ A minister can announce new treatment access in a sentence; it is harder to explain procurement rules, planning controls, marketing restrictions, reformulation targets, active travel or access to safe green space. This difference in political visibility matters because what is easiest to announce can become what is easiest to fund.

## What primary care inherits

Primary care sits across the obesity continuum. In clinical reality, GLP-1 prescribing is not simply a decision to start an injection. It requires assessment of eligibility, expectations, contraindications, dose escalation, side effects, nutrition, muscle preservation, drug interactions, pregnancy advice, diabetes medicines, mental health context, safeguarding concerns, monitoring, discontinuation and reinitiation. Older adults, people with frailty risk and people with multimorbidity need particular attention because rapid weight loss can include lean mass loss and functional harm unless treatment is supported by nutrition and resistance exercise advice.^13^ Routine care is also less controlled than trials. Patients may stop because of cost, supply, intolerance, administrative barriers or anxiety, then return requesting restart after weight regain. Real-world discontinuation and reinitiation are already common.^14^

This creates a practical paradox. A drug that improves individual outcomes can still increase system workload if it is introduced without dietetic capacity, continuity and clear pathways. Starting treatment slowly, preventing unnecessarily rapid weight loss, reviewing medicines and giving realistic activity advice are part of safe care. For patients with joint pain or limited mobility, that advice may need to include low-impact options such as walking, cycling or e-biking, water-based activity, physiotherapy-supported movement or simple resistance activity at home. Primary care may become the place where society manages the metabolic effects of environments it has chosen not to regulate. The question for community health is not whether GLP-1 medicines should be used, but whether their use is organised as part of comprehensive obesity care.

The burden is also relational. Patients may arrive hopeful, ashamed, sceptical, privately self-treating or exhausted by years of advice that felt moralising and ineffective. A good consultation has to validate obesity as a disease while also explaining that medication is not a short course. It has to make room for uncertainty: whether the patient will tolerate escalation, whether treatment will be funded, what happens during shortages, and how progress will be judged beyond the scale. These are primary care tasks because they depend on continuity and trust, not simply prescribing authority.

## Obesogenic environments cannot be prescribed away

The food environment shapes what is available, affordable, promoted, convenient and normalised. Recent reviews link neighbourhood and consumer food environments with dietary intake and obesity-related outcomes, while umbrella reviews associate ultra-processed food exposure with a wide range of adverse health outcomes.^15-18^ The wider physical environment also matters: transport, urban design, public space, safety, work patterns and household resources shape whether regular activity is realistic.^33^ These exposures begin long before a person enters an obesity clinic or asks a GP about medication. A prescription can reduce appetite, weight and risk after years of exposure; it cannot make school meals healthier, change supermarket promotions, reduce the density of unhealthy outlets, make minimally processed food affordable, or prevent children from growing up surrounded by engineered, heavily marketed products.

Population prevention acts earlier in the causal chain. School food interventions, sugar-sweetened beverage taxes, fiscal measures on unhealthy products, front-of-pack labelling, warning labels, reformulation, active-travel policies, green-space planning and Food-EPI-style policy benchmarking all aim to reduce exposure before disease eligibility occurs.^19-25,33^ Food-EPI-style benchmarking means structured assessment of food-environment policy against good-practice indicators, so that gaps in policy strength, coverage and implementation can be made visible. They are less dramatic than a large weight-loss graph, but their purpose is broader: to shift defaults for everyone, including people who will never seek medical care. The public health dividend is therefore not only lower mean weight; it includes better diet quality, reduced cardiometabolic risk, improved oral health, lower fatty liver risk and narrower health inequalities.

This distinction is especially important for children and young people. Childhood exposure to unhealthy food marketing, poor school food, large portions, cheap energy-dense products and inactive local environments normalises risk before autonomy is fully developed. To rely mainly on future pharmacotherapy is to accept a long delay between preventable exposure and clinical response. A credible community health strategy should shorten that delay by reducing harmful defaults early, not by waiting until disease severity justifies a prescription.

## The Integrated Obesity Response Framework

The Integrated Obesity Response Framework starts from a simple premise: obesity requires one coordinated response, not a contest between prevention and treatment. The framework has three complementary pillars: population-level prevention, targeted prevention and early risk management for people at increased risk, and comprehensive treatment for people living with obesity. Primary care contributes across all three, while public health, education, transport, planning, fiscal policy and food systems determine whether the wider environment makes healthier choices possible.^32^ Figure 1 shows how these components interact through clinical, community and policy routes.

**Figure 1.**
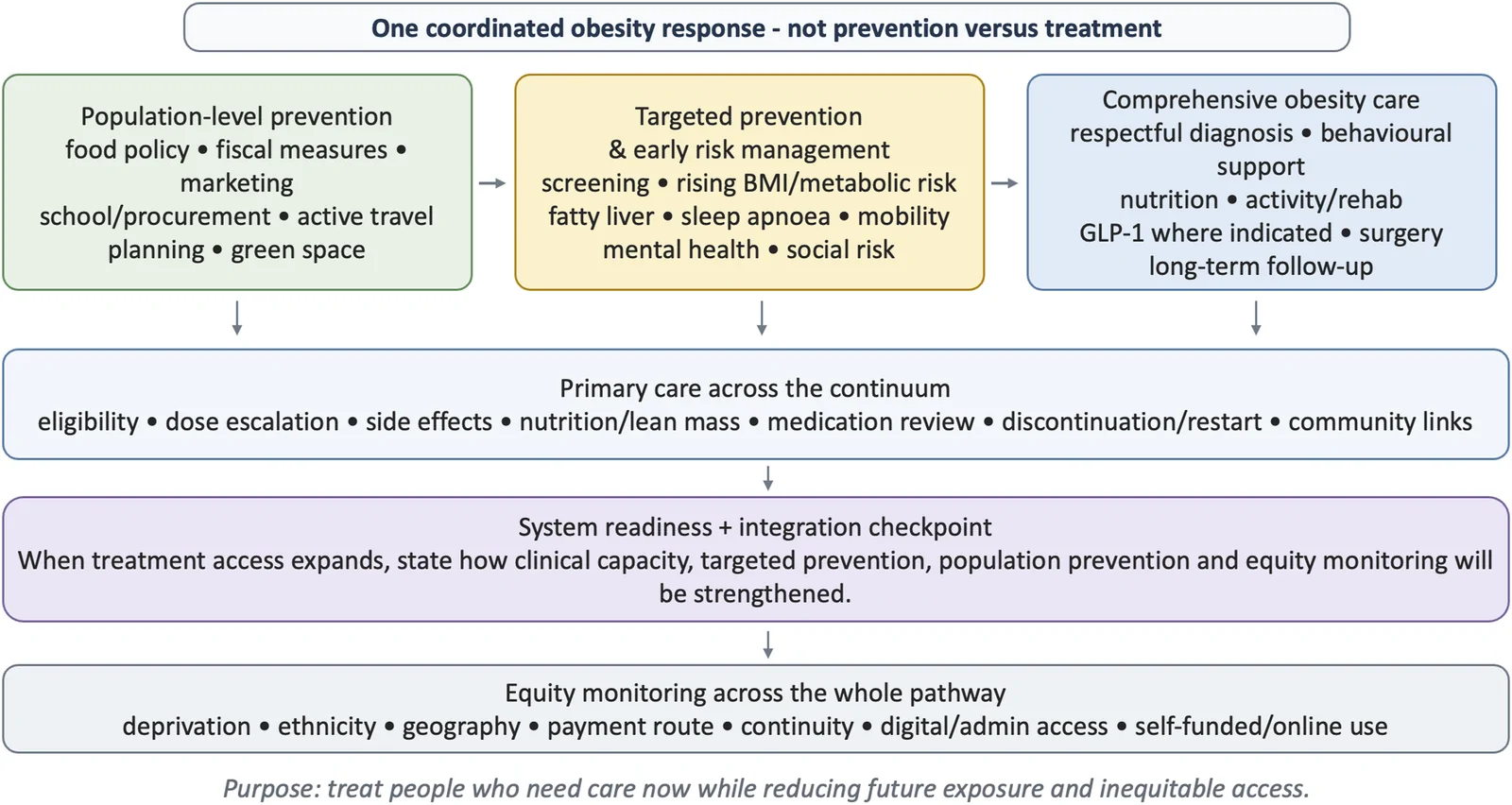
The Integrated Obesity Response Framework for GLP-1-based obesity care in primary care and community health. The framework shows obesity policy as one coordinated response across population-level prevention, targeted prevention and early risk management, and comprehensive obesity care. Obesogenic exposure pressure is reduced through food, activity, transport, planning and commercial-determinant policies. Primary care contributes across the continuum but needs system readiness, workforce support and clear pathways. The integration checkpoint asks whether each expansion in pharmacotherapy access is accompanied by clinical capacity, targeted prevention, population prevention and equity monitoring, rather than allowing treatment volume to become the only visible indicator of action

Population-level prevention reduces obesogenic exposure pressure. This includes action on product formulation, price, marketing, portion size, school and workplace food standards, public procurement, outlet density, online delivery, local planning, active transport and access to public spaces for physical activity. Exposure pressure is not experienced equally. It is usually strongest where poverty, insecure work, poor housing, limited cooking facilities, unsafe streets and fewer healthy retail options overlap. Reducing this pressure should lower future incident obesity and metabolic risk, although effects will take time to appear.

Targeted prevention and early risk management sit between population policy and established disease care. It includes early identification of weight gain, prediabetes, hypertension, fatty liver risk, sleep apnoea, mobility limitation, mental health distress, medication-related weight gain and family or social risk. This is where primary care can intervene before complications are advanced, using brief advice when appropriate but also more structured support, referral, medication review and follow-up when risk is rising.

Comprehensive obesity care is the treatment pillar. It includes respectful diagnosis, shared decision-making, behavioural support, therapeutic nutrition, physical activity or rehabilitation support, pharmacotherapy where indicated, metabolic surgery where appropriate, long-term review and management of obesity-related complications. GLP-1-based therapies belong in this pillar. Their value is not diminished by saying that they are not population prevention; they are essential clinical tools for people who need treatment.

Primary care implementation workload grows as pharmacotherapy expands. This includes counselling patients who are ineligible, supporting those who are eligible, managing adverse effects, reviewing diabetes and antihypertensive medicines, identifying nutritional compromise, advising on resistance activity, managing supply interruptions, documenting outcomes and responding to weight regain after stopping. This workload is not a side issue; it is where policy becomes practice. If primary care capacity is not funded, drug expansion may create inequitable access and unsafe variability even when medicines are clinically effective.

The framework also includes an integration checkpoint. This is not a gate that delays treatment. It is a planning discipline: whenever treatment access expands, decision-makers should state how clinical capacity, targeted prevention, population prevention and equity monitoring will be strengthened at the same time. A health system that funds medicines but leaves pathways, workforce and obesogenic exposures untouched has not built a comprehensive obesity strategy; it has built a treatment channel.

Equity monitoring should assess the risk that benefits follow money, administrative capacity and continuity rather than need. Expensive medicines are prone to unequal uptake, and cost-effectiveness studies show that broad use at current prices may strain public budgets.^26-28^ Access and dispensed prescriptions are already patterned by insurance, social vulnerability, ethnicity and geography.^29-31^ Inequalities may widen when exposure pressure remains high in deprived communities while treatment access is easier for people with stronger advocacy, coverage or private purchasing power.

The framework distinguishes between an integrated response and a treatment-only response. In an integrated response, GLP-1 access expands alongside primary care capacity, targeted prevention, population-level prevention and equity monitoring. Treated patients benefit, access is monitored and future exposure is reduced. In a treatment-only response, individual outcomes may improve for those who obtain and tolerate treatment, but new cases continue to arise, primary care absorbs rising demand, and the commercial and environmental determinants of obesity remain largely unchanged.

This framework generates five propositions. First, indicated GLP-1 prescribing can benefit individual patients and should not be delayed until prevention policy is perfect. Second, treatment expansion alone will not reduce future incident obesity while obesogenic exposure pressure remains high. Third, primary care workload will rise fastest where drug pathways lack dietetic input, activity support, medication-review capacity and restart guidance. Fourth, inequalities will widen unless treatment initiation, dispensed prescriptions, persistence, discontinuation, outcomes, self-funded use and environmental exposure are monitored by social position and geography. Fifth, the most sustainable obesity strategy will be the one that treats patients well today while reducing future need for long-term treatment.

The framework also changes the language of accountability. Instead of asking only whether a country has made GLP-1 medicines available, policymakers should ask whether that access sits within a coherent obesity strategy. For example, a decision to fund wider pharmacotherapy could be accompanied by mandatory local reporting of unhealthy food outlet density, school food compliance, sugary drink purchases, front-of-pack labelling adoption, procurement standards and obesity-treatment access by deprivation quintile. These indicators would not prove causality alone, but they would show whether treatment has become part of a wider response or the only response being measured.

The Integrated Obesity Response Framework can be used prospectively or retrospectively. Prospectively, it can guide impact assessments before a new obesity pharmacotherapy pathway is commissioned. Decision-makers would have to state which prevention actions will accompany clinical expansion, which inequalities will be monitored, and what primary care capacity is required. Retrospectively, it can be used to examine whether prescribing growth has been accompanied by reduced exposure or only by greater clinical activity. In both uses, the central outcome is not simply weight loss among treated patients. It is whether the system is meeting current need while reducing future preventable harm.

The framework also recognises time lag. Treatment benefit is fast, visible and politically attractive; exposure reduction is slower, distributed and easier to neglect. That asymmetry is exactly why integration has to be explicit. Without an integration checkpoint, the intervention that produces the quickest measurable outcome will dominate budget meetings, media narratives and ministerial announcements. The checkpoint forces a second question whenever therapeutic expansion is celebrated: what has been strengthened around the patient, in primary care and upstream? This question protects clinicians from carrying an impossible burden alone and protects prevention from being displaced by the success of modern pharmacology.

## Implications for primary care and community health

For primary care, the framework supports an integrated position. Clinicians should not be asked to choose between compassion for patients and criticism of obesogenic systems. Both are necessary. At consultation level, GLP-1 medicines require careful assessment, shared decision-making, review of comorbidities, attention to nutrition and honest discussion about long-term use and weight regain. At system level, practices and primary care networks need pathways that define who initiates treatment, who monitors safety, how side effects are managed, when medicines are stopped, how restart is handled and how dietetic or community support is accessed.

For community health and commissioning, the integration checkpoint creates a practical test. Any local or national expansion of pharmacotherapy should be accompanied by a prevention plan with measurable exposure indicators. Commissioners could track not only treatment initiations, persistence and weight change, but also inequalities in access, adverse-event workload, discontinuation, restart requests and referral pressure. Public health teams could report whether school food, retail planning, procurement, labelling and fiscal policies are moving in the same direction as treatment expansion. The aim is not to slow access for patients; it is to stop access becoming the only visible action.

Research could then move beyond the familiar treatment-versus-prevention debate. Comparative studies could examine areas with similar prescribing access but different food-environment action, or areas with similar prevention policies but different clinical capacity. Qualitative work could explore how patients and clinicians experience integrated or fragmented pathways. Economic analysis could ask whether coordinated prevention improves the long-term value of pharmacotherapy by reducing new demand.

## A better test of progress

That standard is demanding, but it is also fair. Patients should not be denied effective medicines while waiting for perfect policy, and clinicians should not be asked to compensate indefinitely for preventable exposure. An integrated response accepts both truths. It treats GLP-1 therapies as serious clinical tools and population prevention as a serious public responsibility, then asks whether treatment, primary care capacity and upstream action are being strengthened together. Without that test, national and local obesity policy risks mistaking the expansion of treatment for the reduction of preventable harm in practice.

The arrival of effective incretin-based therapies is a clinical advance and should be treated as such. But a prescription is not a prevention policy. If health systems celebrate treatment while leaving exposure, targeted prevention and primary care capacity weak, they will convert obesity into an expanding channel for long-term downstream care. The better test of progress is whether fewer people are pushed towards needing that care, and whether those who already need treatment can receive it safely and fairly. GLP-1 medicines should therefore mark the beginning of a more serious obesity settlement, not the end of one. Obesity policy has succeeded only when primary care is supported to care for patients today and society is made less likely to produce the same harm tomorrow.
